# On Complexity of Deterministic and Nondeterministic Decision Trees for Conventional Decision Tables from Closed Classes

**DOI:** 10.3390/e25101411

**Published:** 2023-10-03

**Authors:** Azimkhon Ostonov, Mikhail Moshkov

**Affiliations:** Computer, Electrical and Mathematical Sciences & Engineering Division and Computational Bioscience Research Center, King Abdullah University of Science and Technology (KAUST), Thuwal 23955-6900, Saudi Arabia; mikhail.moshkov@kaust.edu.sa

**Keywords:** closed classes of decision tables, deterministic decision trees, nondeterministic decision trees

## Abstract

In this paper, we consider classes of conventional decision tables closed relative to the removal of attributes (columns) and changing decisions assigned to rows. For tables from an arbitrary closed class, we study the dependence of the minimum complexity of deterministic and nondeterministic decision trees on the complexity of the set of attributes attached to columns. We also study the dependence of the minimum complexity of deterministic decision trees on the minimum complexity of nondeterministic decision trees. Note that a nondeterministic decision tree can be interpreted as a set of true decision rules that covers all rows of the table.

## 1. Introduction

Decision tables (sometimes represented as datasets or finite information systems with a distinguished decision attribute) appear in data analysis [1,2,3,4,5,6] and in such areas as combinatorial optimization, computational geometry, and fault diagnosis, where they are used to represent and explore problems [7,8].

Decision trees [1,5,6,7,9] and decision rule systems [2,3,4,8,10,11,12] are widely used as classifiers, as a means for knowledge representation, and as algorithms for solving various problems of combinatorial optimization, fault diagnosis, etc. Decision trees and rules are among the most interpretable models in data analysis [13].

In this paper, we consider classes of conventional decision tables closed under the removal of columns and changing of decisions. The most natural examples of such classes are closed classes of decision tables generated by information systems (see Section 3.4 for an explanation). We study the dependence of the minimum complexity of deterministic and nondeterministic decision trees on the complexity of the set of attributes attached to columns of the decision table. We also study the dependence of the minimum complexity of deterministic decision trees on the minimum complexity of nondeterministic decision trees. Note that the nondeterministic decision trees can be considered as representations of systems of true decision rules that cover all rows of decision tables. Note also that the depth of deterministic and nondeterministic decision trees for computation Boolean functions was studied quite intensively [14,15,16,17].

This paper continues the study of closed classes of decision tables that began with work [18] and continued with works [19,20]. To the best of our knowledge, there are no other papers that study the closed classes of decision tables.

Various classes of objects that are closed under different operations are intensively studied. Among them, in particular, are classes of Boolean functions closed under the operation of superposition [21] and minor-closed classes of graphs [22]. Decision tables represent an interesting and important mathematical object deserving of mathematical research, in particular, the study of closed classes of decision tables.

In [18], we studied the dependence of the minimum depth of deterministic decision trees and the depth of deterministic decision trees constructed by a greedy algorithm on the number of attributes (columns) for conventional decision tables from classes closed under operations of removal of columns and changing of decisions.

In [19], we considered classes of decision tables with many-valued decisions closed under operations of removal of columns, changing of decisions, permutation of columns, and duplication of columns. We studied relationships among three parameters of these tables: the complexity of a decision table (if we consider the depth of decision trees, then the complexity of a decision table is the number of columns in it), the minimum complexity of a deterministic decision tree, and the minimum complexity of a nondeterministic decision tree. We considered a rough classification of functions characterizing relationships and enumerated all possible seven types of relationships.

In [20], we considered classes of decision tables with 0–1 decisions (each row is labeled with the decision 0 or the decision 1) closed relative to the removal of attributes (columns) and changing decisions assigned to rows. For tables from an arbitrary closed class, we studied the dependence of the minimum complexity of deterministic decision trees on various parameters of the tables: the minimum complexity of a test, the complexity of the set of attributes attached to columns, and the minimum complexity of a strongly nondeterministic decision tree. We also studied the dependence of the minimum complexity of strongly nondeterministic decision trees on the complexity of the set of attributes attached to columns. Note that a strongly nondeterministic decision tree can be interpreted as a set of true decision rules that covers all rows labeled with the decision 1.

In the previous papers, we did not consider in detail conventional decision tables in which rows are labeled with arbitrary decisions. These tables differ significantly from tables with many-valued decisions and from tables with 0–1 decisions considered previously. We now describe the results obtained in the present paper. Let *A* be a class of conventional decision tables closed under the removal of columns and changing of decisions, and let ψ be a bounded complexity measure. In this paper, we study three functions: $F_{\psi ,A}\left(n\right)$, $G_{\psi ,A}\left(n\right)$ and $H_{\psi ,A}\left(n\right)$.

The function $F_{\psi ,A}\left(n\right)$ characterizes the growth in the worst case of the minimum complexity of a deterministic decision tree for a decision table from *A* with the growth of the complexity of the set of attributes attached to columns of the table. We prove that the function $F_{\psi ,A}\left(n\right)$ is either bounded from above by a constant or grows as a logarithm of *n*, or it grows almost linearly depending on *n* (it is bounded from above by *n* and is equal to *n* for infinitely many *n*). These results are generalizations of results obtained in [20] for closed classes of decision tables with 0–1 decisions.

The function $G_{\psi ,A}\left(n\right)$ characterizes the growth in the worst case of the minimum complexity of a nondeterministic decision tree for a decision table from *A* with the growth of the complexity of the set of attributes attached to columns of the table. We prove that the function $G_{\psi ,A}\left(n\right)$ is either bounded from above by a constant or grows almost linearly depending on *n* (it is bounded from above by *n* and is equal to *n* for infinitely many *n*).

The function $H_{\psi ,A}\left(n\right)$ characterizes the growth in the worst case of the minimum complexity of a deterministic decision tree for a decision table from *A* with the growth of the minimum complexity of a nondeterministic decision tree for the table. This function is either not everywhere defined or is everywhere defined. Let $H_{\psi ,A}\left(n\right)$ be everywhere defined. We proved that this function is either bounded from above by a constant or it is greater than or equal to *n* for infinitely many *n*.

The novelty of the work is as follows:

- For the function $F_{\psi ,A}$, which characterizes the complexity of deterministic decision trees, we have received an exhaustive description of the types of its behavior.
- For the function $G_{\psi ,A}$, which characterizes the complexity of nondeterministic decision trees, we have received an exhaustive description of the types of its behavior.
- For the function $H_{\psi ,A}$, which characterizes relationships between the complexity of deterministic and nondeterministic decision trees, we have received a preliminary description of the types of its behavior that requires additional study.

The obtained results allow us to point out the cases when the complexity of deterministic and nondeterministic decision trees is essentially less than the complexity of the set of attributes attached to columns of the table. This may be useful in applications.

The paper consists of six sections. In Section 2, main definitions and notation are considered. In Section 3, we provide the main results. Section 4 contains auxiliary statements. In Section 5, we prove the main results. Section 6 contains short conclusions.

## 2. Main Definitions and Notation

Denote ω={0,1,2,…} and, for any k∈ω\{0,1}, denote $E_{k}=\left\{0,1,\ldots ,k-1\right\}$. Let $P=\{f_{i}:i\in \omega \}$ be the set of *attributes* (really names of attributes). Two attributes $f_{i},f_{j}\in P$ are considered different if i≠j.

### 2.1. Decision Tables

First, we define the notion of a decision table.

**Definition** **1.** *Let k∈ω\{0,1}. Denote by $M_{k}$ the set of rectangular tables filled with numbers from $E_{k}$ in each of which rows are pairwise different, each row is labeled with a number from ω (decision), and columns are labeled with pairwise different attributes from P. Rows are interpreted as tuples of values of these attributes. Empty tables without rows belong also to the set $M_{k}$. We will use the same notation* Λ *for these tables. Tables from $M_{k}$ will be called* decision tables.

**Figure 1:**
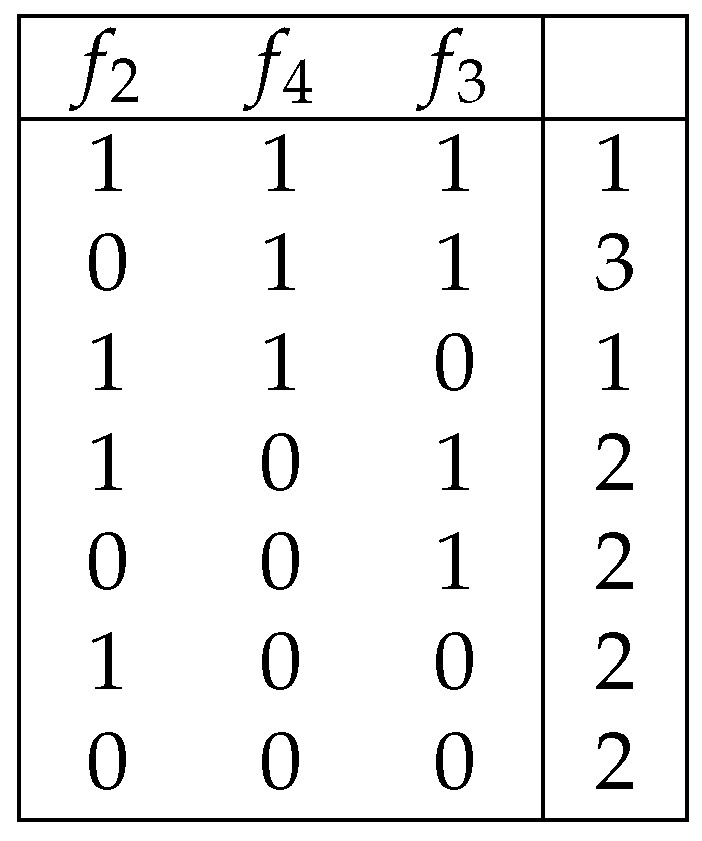
Decision table from $M_{2}$.

**Example** **1.** *Figure 1 shows a decision table from $M_{2}$.*

Denote by $M_{k}C$ the set of tables from $M_{k}$ in each of which all rows are labeled with the same decision. Let Λ∈$M_{k}C$.

Let *T* be a nonempty table from $M_{k}$. Denote by P(T) the set of attributes attached to columns of the table *T*. Let $f_{i_{1}},\ldots ,f_{i_{m}}\in P\left(T\right)$ and $\delta_{1},\ldots ,\delta_{m}\in E_{k}$. We denote by $T\left(f_{i_{1}},\delta_{1}\right)\cdots \left(f_{i_{m}},\delta_{m}\right)$ the table obtained from *T* by the removal of all rows that do not satisfy the following condition: in columns labeled with attributes $f_{i_{1}},\ldots ,f_{i_{m}}$, the row has numbers $\delta_{1},\ldots ,\delta_{m}$, respectively.

We now define two operations on decision tables: the removal of columns and changing of decisions. Let $T\in M_{k}$.

**Definition** **2.** Removal of columns. *Let D⊆P(T). We remove from T all columns labeled with the attributes from the set D. In each group of equal rows on the remaining columns, we keep one with the minimum decision. Denote the obtained table by I(D,T). In particular, I(∅,T)=T and I(P(T),T)=Λ. It is obvious that I(D,T)∈$M_{k}$.*

**Definition** **3.** Changing of decisions. *Let $\nu :E_{k}^{\left|P(T)\right|}\to \omega$ (by definition, $E_{k}^{0}=\emptyset$). For each row $\bar{\delta }$ of the table T, we replace the decision attached to this row with $\nu (\bar{\delta })$. We denote the obtained table by J(ν,T). It is obvious that J(ν,T)∈$M_{k}$.*

**Definition** **4.** *Denote $\left[T\right]=\left\{J\left(\nu ,I\left(D,T\right)\right):D\subseteq P\left(T\right),\nu :E_{k}^{\left|P(T)\backslash D\right|}\to \omega \right\}$. The set $\left[T\right]$ is the* closure of the table *T under the operations of removal of columns and changing of decisions.*

**Figure 2:**
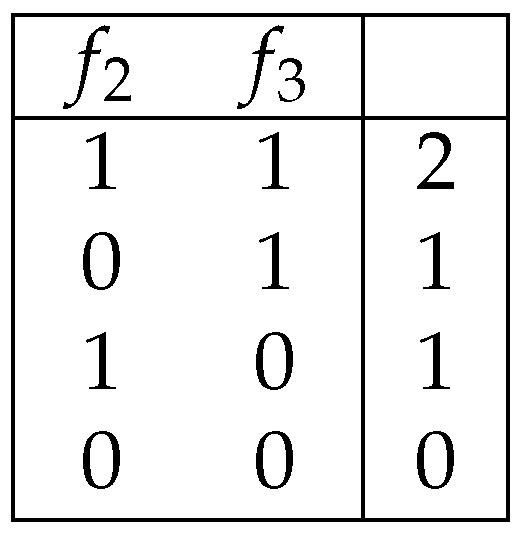
Decision table obtained from the decision table shown in Figure 1 by removal of a column and changing of decisions.

**Example** **2.** *Figure 2 shows the table $J(\nu ,I\left(D,T_{0}\right))$, where $T_{0}$ is the table shown in Figure 1, $D=\{f_{4}\}$ and $\nu \left(x_{1},x_{2}\right)=x_{1}+x_{2}$.*

**Definition** **5.** *Let $A\subseteq M_{k}$ and A≠∅. Denote $\left[A\right]={⋃}_{T\in A}\left[T\right]$. The set $\left[A\right]$ is the* closure of the set *A under the considered two operations. The class (the set) of decision tables A will be called a* closed class *if $\left[A\right]=A$.*

Let $A_{1}$ and $A_{2}$ be closed classes of decision tables from $M_{k}$. Then, $A_{1}\cup A_{2}$ is a closed class of decision tables from $M_{k}$.

### 2.2. Deterministic and Nondeterministic Decision Trees

A *finite tree with the root* is a finite directed tree in which exactly one node called the *root* has no entering edges. The nodes without leaving edges are called *terminal* nodes.

**Definition** **6.** *A k*-decision tree *is a finite tree with the root, which has at least two nodes and in which*

- *The root and edges leaving the root are not labeled.*
- *Each terminal node is labeled with a decision from the set ω.*
- *Each node, which is neither the root nor a terminal node, is labeled with an attribute from the set P. Each edge leaving such a node is labeled with a number from the set $E_{k}$.*

**Figure 3:**
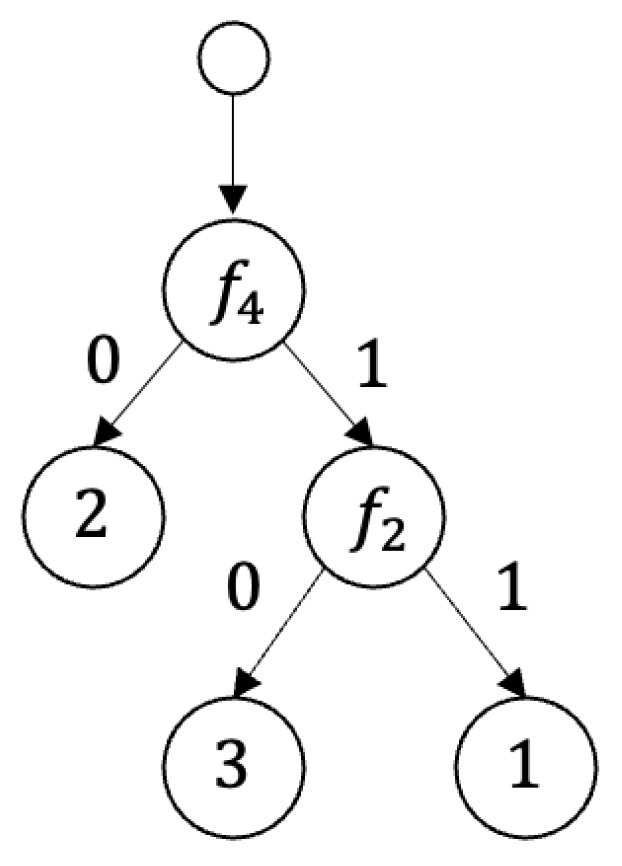
A deterministic decision tree for the decision table shown in Figure 1.

**Figure 4:**
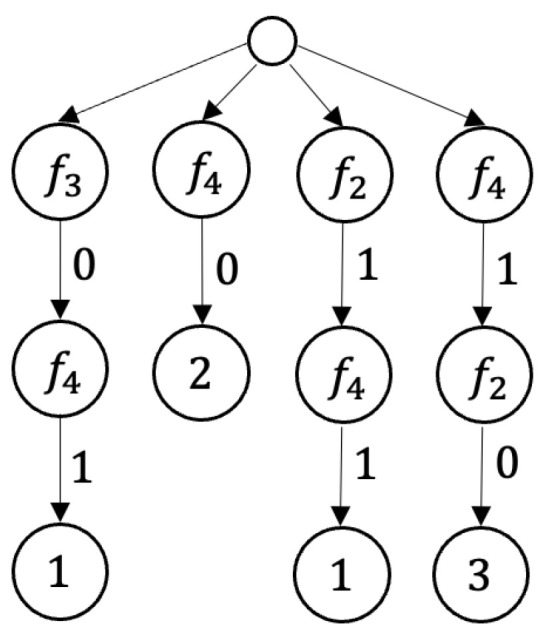
A nondeterministic decision tree for the decision table shown in Figure 1.

**Example** **3.** *Figure 3 and Figure 4 show 2-decision trees.*

We denote by $T_{k}$ the set of all *k*-decision trees. Let $\Gamma \in T_{k}$. We denote by P(Γ) the set of attributes attached to nodes of Γ that are neither the root nor terminal nodes. A *complete path* of Γ is a sequence $\tau =v_{1},d_{1},\ldots ,v_{m},d_{m},v_{m+1}$ of nodes and edges of Γ in which $v_{1}$ is the root of Γ, $v_{m+1}$ is a terminal node of Γ and, for j=1,…,m, the edge $d_{j}$ leaves the node $v_{j}$ and enters the node $v_{j+1}$. Let T∈$M_{k}$. If P(Γ)⊆P(T), then we correspond to the table *T* and the complete path τ a decision table T(τ). If m=1, then T(τ)=T. If m>1 and, for j=2,…,m, the node $v_{j}$ is labeled with the attribute $f_{i_{j}}$ and the edge $d_{j}$ is labeled with the number $\delta_{j}$, then $T\left(\tau \right)=T\left(f_{i_{2}},\delta_{2}\right)\cdots \left(f_{i_{m}},\delta_{m}\right)$.

**Definition** **7.** *Let $T\in M_{k}\backslash \left\{\Lambda \right\}$. A* deterministic decision tree *for the table T is a k-decision tree* Γ *satisfying the following conditions:*

- *Only one edge leaves the root of* Γ.
- *For any node, which is neither the root nor a terminal node, edges leaving this node are labeled with pairwise different numbers.*
- *P(Γ)⊆P(T).*
- *For any row of T, there exists a complete path τ of* Γ *such that the considered row belongs to the table T(τ).*
- *For any complete path τ of* Γ, *either T(τ)=Λ or all rows of T(τ) are labeled with the decision attached to the terminal node of τ.*

**Example** **4.** *The* 2-*decision tree shown in Figure 3 is a deterministic decision tree for the decision table shown in Figure 1.*

**Definition** **8.** *Let T∈$M_{k}\backslash \left\{\Lambda \right\}$. A* nondeterministic decision tree *for the table T is a k-decision tree* Γ *satisfying the following conditions:*

- *P(Γ)⊆P(T).*
- *For any row of T, there exists a complete path τ of* Γ *such that the considered row belongs to the table T(τ).*
- *For any complete path τ of* Γ, *either T(τ)=Λ or all rows of T(τ) are labeled with the decision attached to the terminal node of τ.*

**Example** **5.** *The* 2-*decision tree shown in Figure 4 is a nondeterministic decision tree for the decision table shown in Figure 1.*

### 2.3. Complexity Measures

Denote by *B* the set of all finite words over the alphabet $P=\{f_{i}:i\in \omega \}$, which contains the empty word λ and on which the word concatenation operation is defined.

**Definition** **9.** *A* complexity measure *is an arbitrary function ψ:B→ω that has the following properties: for any words $\alpha_{1},\alpha_{2}\in B$,*

- *$\psi (\alpha_{1})=0$ if and only if $\alpha_{1}=\lambda$—*positivity *property.*
- *$\psi \left(\alpha_{1}\right)=\psi \left(\alpha_{1}^{\prime }\right)$ for any word $\alpha_{1}^{\prime }$ obtained from $\alpha_{1}$ by permutation of letters—*commutativity *property.*
- *$\psi \left(\alpha_{1}\right)\leq \psi \left(\alpha_{1}\alpha_{2}\right)$—*nondecreasing *property.*
- *$\psi \left(\alpha_{1}\alpha_{2}\right)\leq \psi \left(\alpha_{1}\right)+\psi \left(\alpha_{2}\right)$—*boundedness from above *property.*

The following functions are complexity measures:

- Function *h* for which, for any word α∈B, $h\left(\alpha \right)=\left|\alpha \right|$, where $\left|\alpha \right|$ is the length of the word α.
- An arbitrary function φ:B→ω such that φ(λ)=0, for any $f_{i}\in P$, $\varphi (f_{i})>0$ and, for any nonempty word $f_{i_{1}}\cdots f_{i_{m}}\in B$,
  (1) $$\varphi \left(f_{i_{1}}\cdots f_{i_{m}}\right)=\sum_{j=1}^{m}\varphi \left(f_{i_{j}}\right).$$
- An arbitrary function ρ:B→ω such that ρ(λ)=0, for any $f_{i}\in P$, $\rho (f_{i})>0$, and, for any nonempty word $f_{i_{1}}\cdots f_{i_{m}}\in B$, $\rho \left(f_{i_{1}}\cdots f_{i_{m}}\right)=\max\left\{\rho \left(f_{i_{j}}\right):j=1,\ldots ,m\right\}$.

**Definition** **10.** *A* bounded *complexity measure is a complexity measure ψ, which has the* boundedness from below *property: for any word α∈B, $\psi \left(\alpha \right)\geq \left|\alpha \right|$.*

Any complexity measure satisfying the equality (1), in particular the function *h*, is a bounded complexity measure. One can show that if functions $\psi_{1}$ and $\psi_{2}$ are complexity measures, then the functions $\psi_{3}$ and $\psi_{4}$ are complexity measures, where for any α∈B, $\psi_{3}\left(\alpha \right)=\psi_{1}\left(\alpha \right)+\psi_{2}\left(\alpha \right)$ and $\psi_{4}\left(\alpha \right)=\max\left(\psi_{1}\left(\alpha \right),\psi_{2}\left(\alpha \right)\right)$. If the function $\psi_{1}$ is a bounded complexity measure, then the functions $\psi_{3}$ and $\psi_{4}$ are bounded complexity measures.

**Definition** **11.** *Let ψ be a complexity measure. We extend it to the set of all finite subsets of the set P. Let D be a finite subset of the set P. If D=∅, then ψ(D)=0. Let $D=\{f_{i_{1}},\ldots ,f_{i_{m}}\}$ and m≥1. Then, $\psi \left(D\right)=\psi \left(f_{i_{1}}\cdots f_{i_{m}}\right)$.*

### 2.4. Parameters of Decision Trees and Tables

Let $\Gamma \in T_{k}$ and $\tau =v_{1},d_{1},\ldots ,v_{m},d_{m},v_{m+1}$ be a complete path of Γ. We correspond to the path τ a word F(τ)∈B: if m=1, then F(τ)=λ, and if m>1 and, for j=2,…,m, the node $v_{j}$ is labeled with the attribute $f_{i_{j}}$, then $F\left(\tau \right)=f_{i_{2}}\cdots f_{i_{m}}$.

**Definition** **12.** *Let ψ be a complexity measure. We extend the function ψ to the set $T_{k}$. Let $\Gamma \in T_{k}$. Then, ψ(Γ)=max{ψ(F(τ))}, where the maximum is taken over all complete paths τ of the decision tree* Γ. *For a given complexity measure ψ, the value ψ(Γ) will be called the* complexity of the decision tree Γ. *The value h(Γ) will be called the* depth *of the decision tree* Γ.

Let ψ be a complexity measure. We now describe the functions $\psi^{d}$, $\psi^{a}$, Sep, $W_{\psi }$, $S_{\psi }$, ${\hat{S}}_{\psi }$, $M_{\psi }$, and *N* defined on the set $M_{k}$ and taking values from the set ω. By definition, the value of each of these functions for Λ is equal to 0. Let $T\in M_{k}\backslash \left\{\Lambda \right\}$.

- $\psi^{d}\left(T\right)=\min\left\{\psi \left(\Gamma \right)\right\}$, where the minimum is taken over all deterministic decision trees Γ for the table *T*.
- $\psi^{a}\left(T\right)=\min\left\{\psi \left(\Gamma \right)\right\}$, where the minimum is taken over all nondeterministic decision trees Γ for the table *T*.
- A set D⊆P(T) is called a *separating set* for the table *T* if the sets of columns labeled with attributes from *D* rows of the table *T* are pairwise different. Then, Sep(T) is the minimum cardinality of a separating set for the table *T*.
- $W_{\psi }\left(T\right)=\psi \left(P\left(T\right)\right)$. This is the complexity of the set of attributes attached to columns of the table *T*.
- Let $\bar{\delta }$ be a row of the table *T*. Denote $S_{\psi }\left(T,\bar{\delta }\right)=\min\left\{\psi \left(D\right)\right\}$, where the minimum is taken over all subsets *D* of the set P(T) such that in the set of columns of *T* labeled with attributes from *D*, the row $\bar{\delta }$ is different from all other rows of the table *T*. Then, $S_{\psi }\left(T\right)=\max\left\{S_{\psi }\left(T,\bar{\delta }\right)\right\}$, where the maximum is taken over all rows $\bar{\delta }$ of the table *T*.
- ${\hat{S}}_{\psi }\left(T\right)=\max\left\{S_{\psi }\left(T^{*}\right):T^{*}\in \left[T\right]\right\}$.
- If $T\in M_{k}C$, then $M_{\psi }\left(T\right)=0$. Let $T\notin M_{k}C$, $\left|P(T)\right|=n$, and columns of the table *T* be labeled with the attributes $f_{t_{1}},\ldots ,f_{t_{n}}$. Let $\bar{\delta }=\left(\delta_{1},\ldots ,\delta_{n}\right)\in E_{k}^{n}$. Denote by $M_{\psi }\left(T,\bar{\delta }\right)$ the minimum number p∈ω for which there exist attributes $f_{t_{i_{1}}},\ldots ,f_{t_{i_{m}}}\in P\left(T\right)$ such that $T\left(f_{t_{i_{1}}},\delta_{i_{1}}\right)\cdots \left(f_{t_{i_{m}}},\delta_{i_{m}}\right)\in M_{k}C$ and $\psi (f_{t_{i_{1}}}\cdots f_{t_{i_{m}}})=p$. Then, $M_{\psi }\left(T\right)=\max\left\{M_{\psi }\left(T,\bar{\delta }\right):\bar{\delta }\in E_{k}^{n}\right\}$.
- N(T) is the number of rows in the table *T*.

For the complexity measure *h*, we denote $W\left(T\right)=W_{h}\left(T\right)$, $S\left(T\right)=S_{h}\left(T\right)$, $\hat{S}\left(T\right)={\hat{S}}_{h}\left(T\right)$, and $M\left(T\right)=M_{h}\left(T\right)$. Note that W(T) is the number of columns in the table *T*.

**Example** **6.** *We denote by $T_{0}$ the decision table shown in Figure 1. One can show that $h^{d}\left(T_{0}\right)=2$, $h^{a}\left(T_{0}\right)=2$, $Sep(T_{0})=3$, $W(T_{0})=3$, $N(T_{0})=7$, $S(T_{0})=3$, $\hat{S}\left(T_{0}\right)=3$, and $M(T_{0})=2$.*

## 3. Main Results

In this section, we consider results obtained for the functions $F_{\psi ,A}$, $G_{\psi ,A}$, and $H_{\psi ,A}$ and discuss closed classes of decision tables generated by information systems.

### 3.1. Function $F_{\psi ,A}$

Let ψ be a bounded complexity measure and *A* be a nonempty closed class of decision tables from $M_{k}$. We now define a function $F_{\psi ,A}:\omega \to \omega$. Let n∈ω. Then

$$F_{\psi ,A}\left(n\right)=\max\left\{\psi^{d}\left(T\right):T\in A,W_{\psi }\left(T\right)\leq n\right\}.$$

The function $F_{\psi ,A}$ characterizes the growth in the worst case of the minimum complexity of a deterministic decision tree for a decision table from *A* with the growth of the complexity of the set of attributes attached to columns of this table.

Let $D=\{n_{i}:i\in \omega \}$ be an infinite subset of the set ω in which, for any i∈ω, $n_{i}<n_{i+1}$. Let us define a function $H_{D}:\omega \to \omega$. Let n∈ω. If $n<n_{0}$, then $H_{D}\left(n\right)=0$. If, for some i∈ω, $n_{i}\leq n<n_{i+1}$, then $H_{D}\left(n\right)=n_{i}$.

**Theorem** **1.** *Let ψ be a bounded complexity measure and A be a nonempty closed class of decision tables from $M_{k}$. Then, $F_{\psi ,A}$ is an everywhere defined nondecreasing function such that $F_{\psi ,A}\left(n\right)\leq n$ for any n∈ω and $F_{\psi ,A}\left(0\right)=0$. For this function, one of the following statements holds:*

*(a)*  *If the functions $S_{\psi }$ and N are bounded from above on class A, then there exists a positive constant $c_{0}$ such that $F_{\psi ,A}\left(n\right)\leq c_{0}$ for any n∈ω.*
*(b)*  *If the function $S_{\psi }$ is bounded from above on class A and the function N is not bounded from above on class A, then there exist positive constants $c_{1}$, $c_{2}$, $c_{3}$, $c_{4}$ such that $c_{1}\log_{2}n-c_{2}\leq F_{\psi ,A}\left(n\right)\leq c_{3}\log_{2}n+c_{4}$ for any n∈ω\{0}.*
*(c)*  *If the function $S_{\psi }$ is not bounded from above on class A, then there exists an infinite subset D of the set ω such that $H_{D}\left(n\right)\leq F_{\psi ,A}\left(n\right)$ for any n∈ω.*

Thus, for the function $F_{\psi ,A}$, we have received an exhaustive description of the types of its behavior. Type (a) is degenerate: the number of rows in decision tables from the closed class is limited from above by a constant. Type (b) is of most interest to us: the complexity of deterministic decision trees behaves in the worst case as the logarithm on the complexity of the set of attributes in the table. Type (c) is not of particular interest: the complexity of deterministic decision trees in the worst case is the same as the complexity of the set of attributes in the table.

### 3.2. Function $G_{\psi ,A}$

Let ψ be a bounded complexity measure and *A* be a nonempty closed class of decision tables from $M_{k}$. We now define a function $G_{\psi ,A}$. Let n∈ω. Then

$$G_{\psi ,A}\left(n\right)=\max\left\{\psi^{a}\left(T\right):T\in A,W_{\psi }\left(T\right)\leq n\right\}.$$

The function $G_{\psi ,A}$ characterizes the growth in the worst case of the minimum complexity of a nondeterministic decision tree for a decision table from *A* with the growth of the complexity of the set of attributes attached to columns of this table.

**Theorem** **2.** *Let ψ be a bounded complexity measure and A be a nonempty closed class of decision tables from $M_{k}$. Then, $G_{\psi ,A}$ is an everywhere defined nondecreasing function such that $G_{\psi ,A}\left(n\right)\leq n$ for any n∈ω and $G_{\psi ,A}\left(0\right)=0$. For this function, one of the following statements holds:*

*(a)*  *If the function $S_{\psi }$ is bounded from above on class A, then there exists a positive constant c such that $G_{\psi ,A}\left(n\right)\leq c$ for any n∈ω.*
*(b)*  *If the function $S_{\psi }$ is not bounded from above on the class A, then there exists an infinite subset D of the set ω such that $H_{D}\left(n\right)\leq G_{\psi ,A}\left(n\right)$ for any n∈ω.*

Thus, for the function $G_{\psi ,A}$, we have received an exhaustive description of the types of its behavior. Type (a) is of most interest to us: the complexity of nondeterministic decision trees is bounded from above by a constant. Type (b) is not of particular interest: the complexity of nondeterministic decision trees in the worst case is the same as the complexity of the set of attributes in the table.

### 3.3. Function $H_{\psi ,A}$

Let ψ be a bounded complexity measure and *A* be a nonempty closed class of decision tables from $M_{k}$. We now define possibly partial function $H_{\psi ,A}:\omega \to \omega$. Let n∈ω. If the set $\left\{\psi^{d}\left(T\right):T\in A,\psi^{a}\left(T\right)\leq n\right\}$ is infinite, then the value $H_{\psi ,A}\left(n\right)$ is undefined. Otherwise, $H_{\psi ,A}\left(n\right)=\max\left\{\psi^{d}\left(T\right):T\in A,\psi^{a}\left(T\right)\leq n\right\}$.

The function $H_{\psi ,A}$ characterizes the growth in the worst case of the minimum complexity of a deterministic decision tree for a decision table from *A* with the growth of the minimum complexity of a nondeterministic decision tree for this table.

**Theorem** **3.** *Let ψ be a bounded complexity measure and A be a nonempty closed class of decision tables from $M_{k}$. Then, $H_{\psi ,A}\left(0\right)=0$ and $H_{\psi ,A}$ is a nondecreasing function in its domain.*

*If $H_{\psi ,A}$ is not an everywhere defined function, then its domain coincides with the set $\left\{n:n\in \omega ,n\leq n_{0}\right\}$ for some $n_{0}\in \omega$.*

*If the function $H_{\psi ,A}$ is everywhere defined, then one of the following statements holds:*

*(a)*  *If the function $\psi^{d}$ is bounded from above on the class A, then there is a nonnegative constant c such that $H_{\psi ,A}\left(n\right)\leq c$ for any n∈ω.*
*(b)*  *If the function $\psi^{d}$ is not bounded from above on the class A, then there exists an infinite subset D of the set ω such that $H_{\psi ,A}\left(n\right)\geq H_{D}\left(n\right)$ for any n∈ω.*

**Remark** **1.** *From Theorem 1, it follows that the function $\psi^{d}$ is bounded from above on class A if and only if the functions $S_{\psi }$ and N are bounded from above on class A.*

For the function $H_{\psi ,A}$, we have received a preliminary description of the types of its behavior. Type (a) is degenerate: the number of rows in decision tables from the closed class is limited from above by a constant. Type (b) is of most interest to us. However, more research is needed to understand how the function can behave within this type.

### 3.4. Family of Closed Classes of Decision Tables

Let *U* be a set and $\Phi =\{f_{0},f_{1},\ldots \}$ be a finite or countable set of functions (attributes) defined on *U* and taking values from $E_{k}$. The pair (U,Φ) is called a *k*-*information system*. A *problem* over (U,Φ) is an arbitrary tuple $z=(U,\nu ,f_{i_{1}},\ldots ,f_{i_{n}})$, where n∈ω\{0}, $\nu :E_{k}^{n}\to \omega$ and $f_{i_{1}},\ldots ,f_{i_{n}}$ are functions from Φ with pairwise different indices $i_{1},\ldots ,i_{n}$. The problem *z* is to determine the value $\nu (f_{i_{1}}\left(u\right),\ldots ,f_{i_{n}}\left(u\right))$ for a given u∈U. Various examples of *k*-information systems and problems over these systems can be found in [7].

We denote by T(z) a decision table from $M_{k}$ with *n* columns labeled with attributes $f_{i_{1}},\ldots ,f_{i_{n}}$. A row $\left(\delta_{1},\ldots ,\delta_{n}\right)\in E_{k}^{n}$ belongs to the table T(z) if and only if the system of equations $\left\{f_{i_{1}}\left(x\right)=\delta_{1},\ldots ,f_{i_{n}}\left(x\right)=\delta_{n}\right\}$ has a solution from the set *U*. This row is labeled with the decision $\nu (\delta_{1},\ldots ,\delta_{n})$.

Let the algorithms for solving problem *z* be algorithms in which each elementary operation consists of calculating the value of some attribute from the set $\left\{f_{i_{1}},\ldots ,f_{i_{n}}\right\}$ on a given element u∈U. Then, as a model of the problem *z*, we can use the decision table T(z), and as models of algorithms for solving the problem *z*, we can use deterministic and nondeterministic decision trees for the table T(z).

Denote by Z(U,Φ) the set of problems over (U,Φ) and A(U,Φ)={T(z):z∈Z(U,Φ)}. One can show that A(U,Φ)=[A(U,Φ)]; i.e., A(U,Φ) is a closed class of decision tables from $M_{k}$ *generated* by the information system (U,Φ).

Closed classes of decision tables generated by *k*-information systems are the most natural examples of closed classes. However, the notion of a closed class is essentially wider. In particular, the union $A\left(U_{1},\Phi_{1}\right)\cup A\left(U_{2},\Phi_{2}\right)$, where $\left(U_{1},\Phi_{1}\right)$ and $\left(U_{2},\Phi_{2}\right)$ are *k*-information systems, is a closed class, but generally, we cannot find an information system (U,Φ) such that $A\left(U,\Phi \right)=A\left(U_{1},\Phi_{1}\right)\cup A\left(U_{2},\Phi_{2}\right)$.

### 3.5. Example of Information System

Let R be the set of real numbers and $F=\{f_{i}:i\in \omega \}$ be the set of functions defined on R and taking values from the set $E_{2}$ such that, for any i∈ω and a∈R,

$$f_{i}\left(a\right)=\left\{\begin{array}{cc} 0, & a<i,\\ 1, & a\geq i. \end{array}\right.$$

Let ψ be a bounded complexity measure and A=A(R,F). One can prove the following statements:

- The function *N* is not bounded from above on the set *A*.
- The function $S_{\psi }$ is bounded from above on the set *A* if and only if there exists a constant $c_{0}>0$ such that $\psi \left(f_{i}\right)\leq c_{0}$ for any i∈ω.
- The function $\psi^{d}$ is not bounded from above on the set *A*.
- The function $H_{\psi ,A}$ is everywhere defined if and only if, for any n∈ω, the set $\left\{f_{i}:i\in \omega ,\psi \left(f_{i}\right)\leq n\right\}$ is finite.

## 4. Auxiliary Statements

This section contains auxiliary statements.

It is not difficult to prove the following upper bound on the minimum complexity of deterministic decision trees for a table.

**Lemma** **1.** *For any complexity measure ψ and any table T from $M_{k}$,*

$$\psi^{d}\left(T\right)\leq W_{\psi }\left(T\right).$$

The notions of a decision table and a deterministic decision tree used in this paper are somewhat different from the corresponding notions used in [23]. Taking into account these differences, it is easy to prove the following statement, which follows almost directly from Lemma 1.3 and Theorem 2.2 from [23].

**Lemma** **2.** *For any complexity measure ψ and any table T from $M_{k}$,*

$$\psi^{d}\left(T\right)\leq \left\{\begin{array}{cc} 0, & M_{\psi }\left(T\right)=0,\\ M_{\psi }\left(T\right)\log_{2}N\left(T\right), & M_{\psi }\left(T\right)\geq 1. \end{array}\right.$$

The following two statements are simple generalizations of similar results obtained in [20] for decision tables with 0–1-decisions. For the sake of completeness, we present their proofs.

**Lemma** **3.** *For any complexity measure ψ and any table T from $M_{k}$,*

$$M_{\psi }\left(T\right)\leq 2{\hat{S}}_{\psi }\left(T\right).$$

**Proof.**  Let $T\in M_{k}C$. Then, $M_{\psi }\left(T\right)=0$. Therefore, $M_{\psi }\left(T\right)\leq 2{\hat{S}}_{\psi }\left(T\right)$.

Let $T\notin M_{k}C$, W(T)=n and $f_{t_{1}},\ldots ,f_{t_{n}}$ be attributes attached to columns of the table *T*. Denote $D=\{f_{i}:f_{i}\in P\left(T\right),\psi \left(f_{i}\right)\leq S_{\psi }\left(T\right)\}$ and $T^{*}=I\left(P\left(T\right)\backslash D,T\right)$. Evidently, $T^{*}\in \left[T\right]$. Taking into account that the function ψ has the nondecreasing property, we obtain that any two rows of *T* are different in columns labeled with attributes from the set *D*. Let for the definiteness, $D=\{f_{t_{1}},\ldots ,f_{t_{m}}\}$.

Let $\bar{\delta }=\left(\delta_{1},\ldots ,\delta_{n}\right)\in E_{k}^{n}$. Let $\left(\delta_{1},\ldots ,\delta_{m}\right)$ be a row of $T^{*}$. Since $T^{*}\in \left[T\right]$, there exist attributes $f_{t_{j_{1}}},\ldots ,f_{t_{j_{s}}}$ of the table $T^{*}$ such that the row $\left(\delta_{1},\ldots ,\delta_{m}\right)$ is different from all other rows of $T^{*}$ in columns labeled with these attributes and $\psi \left(f_{t_{j_{1}}}\cdots f_{t_{j_{s}}}\right)\leq {\hat{S}}_{\psi }\left(T\right)$. It is clear that $T\left(f_{t_{j_{1}}},\delta_{j_{1}}\right)\cdots \left(f_{t_{j_{s}}},\delta_{j_{s}}\right)\in M_{k}C$. Therefore, $M_{\psi }\left(T,\bar{\delta }\right)\leq {\hat{S}}_{\psi }\left(T\right)$.

Let $\left(\delta_{1},\ldots ,\delta_{m}\right)$ be not a row of $T^{*}$. We consider *m* tables $T^{*}\left(f_{t_{1}},\delta_{1}\right)$, $T^{*}\left(f_{t_{1}},\delta_{1}\right)\left(f_{t_{2}},\delta_{2}\right)$, ⋯, $T^{*}\left(f_{t_{1}},\delta_{1}\right)\cdots \left(f_{t_{m}},\delta_{m}\right)$. If $T^{*}\left(f_{t_{1}},\delta_{1}\right)=\Lambda$, then $T(f_{t_{1}},\delta_{1})=\Lambda$. Since $f_{t_{1}}\in D$, $\psi \left(f_{t_{1}}\right)\leq S_{\psi }\left(T\right)$. Taking into account that $S_{\psi }\left(T\right)\leq {\hat{S}}_{\psi }\left(T\right)$, we obtain $M_{\psi }\left(T,\bar{\delta }\right)\leq {\hat{S}}_{\psi }\left(T\right)$. Let $T^{*}\left(f_{t_{1}},\delta_{1}\right)\neq \Lambda$. Then, there exists p∈{1,…,m−1} such that $T^{*}\left(f_{t_{1}},\delta_{1}\right)\cdots \left(f_{t_{p}},\delta_{p}\right)\neq \Lambda$ and $T^{*}\left(f_{t_{1}},\delta_{1}\right)\cdots \left(f_{t_{p+1}},\delta_{p+1}\right)=\Lambda$. Denote $C=\{f_{t_{p+1}},f_{t_{p+2}},\ldots ,f_{t_{n}}\}$ and $T^{0}=I\left(C,T\right)$. Evidently, $T^{0}\in \left[T\right]$. Therefore, in $T^{0}$, there are attributes $f_{t_{i_{1}}},\ldots ,f_{t_{i_{l}}}$ such that the row $\left(\delta_{1},\ldots ,\delta_{p}\right)$ is different from all other rows of the table $T^{0}$ in columns labeled with these attributes and $\psi \left(f_{t_{i_{1}}}\cdots f_{t_{i_{l}}}\right)\leq {\hat{S}}_{\psi }\left(T\right)$. One can show that

$$T^{*}\left(f_{t_{i_{1}}},\delta_{i_{1}}\right)\cdots \left(f_{t_{i_{l}}},\delta_{i_{l}}\right)=T^{*}\left(f_{t_{1}},\delta_{1}\right)\cdots \left(f_{t_{p}},\delta_{p}\right).$$

Therefore, $T^{*}\left(f_{t_{i_{1}}},\delta_{i_{1}}\right)\cdots \left(f_{t_{i_{l}}},\delta_{i_{l}}\right)\left(f_{t_{p+1}},\delta_{p+1}\right)=\Lambda$. Hence,

$$T\left(f_{t_{i_{1}}},\delta_{i_{1}}\right)\cdots \left(f_{t_{i_{l}}},\delta_{i_{l}}\right)\left(f_{t_{p+1}},\delta_{p+1}\right)=\Lambda .$$

Since $f_{t_{p+1}}\in D$, $\psi \left(f_{t_{p+1}}\right)\leq S_{\psi }\left(T\right)\leq {\hat{S}}_{\psi }\left(T\right)$. Using the boundedness from above property of the function ψ, we obtain $\psi \left(f_{t_{i_{1}}}\cdots f_{t_{i_{l}}}f_{t_{p+1}}\right)\leq 2{\hat{S}}_{\psi }\left(T\right)$. Therefore, $M_{\psi }\left(T,\bar{\delta }\right)\leq 2{\hat{S}}_{\psi }\left(T\right)$.

Thus, for any $\bar{\delta }\in E_{k}^{n}$, $M_{\psi }\left(T,\bar{\delta }\right)\leq 2{\hat{S}}_{\psi }\left(T\right)$. As a result, we obtain $M_{\psi }\left(T\right)\leq 2{\hat{S}}_{\psi }\left(T\right)$.    □

**Lemma** **4.** *For any table T from $M_{k}\backslash \left\{\Lambda \right\}$,*

$$N\left(T\right)\leq {\left(kW\left(T\right)\right)}^{S(T)}.$$

**Proof.**  If N(T)=1, then S(T)=0 and the considered inequality holds. Let N(T)>1. Then, S(T)>0. Denote m=S(T). Evidently, for any row $\bar{\delta }$ of the table *T*, there exist attributes $f_{i_{1}},\ldots ,f_{i_{m}}\in P\left(T\right)$ and numbers $\sigma_{1},\ldots ,\sigma_{m}\in E_{k}$ such that the table $T\left(f_{i_{1}},\sigma_{1}\right)\cdots \left(f_{i_{m}},\sigma_{m}\right)$ contains only the row $\bar{\delta }$. Therefore, there is a one-to-one mapping of rows of the table *T* onto some set *G* of pairs of tuples of the kind $\left(\left(f_{i_{1}},\ldots ,f_{i_{m}}\right),\left(\sigma_{1},\ldots ,\sigma_{m}\right)\right)$ where $f_{i_{1}},\ldots ,f_{i_{m}}\in P\left(T\right)$ and $\sigma_{1},\ldots ,\sigma_{m}\in E_{k}$. Evidently, $\left|G\right|\leq W{\left(T\right)}^{m}k^{m}$. Therefore, $N\left(T\right)\leq {\left(kW\left(T\right)\right)}^{S(T)}$.    □

**Lemma** **5.** *For any table T from $M_{k}\backslash \left\{\Lambda \right\}$, there exists a mapping $\nu :E_{k}^{W(T)}\to \omega$ such that*

$$h^{d}\left(J\left(\nu ,T\right)\right)\geq \log_{k}N\left(T\right).$$

**Proof.**  Let $T\in M_{k}\backslash \left\{\Lambda \right\}$ and $\nu :E_{k}^{W(T)}\to \omega$ be a mapping for which $\nu \left(\bar{\delta }\right)\neq \nu \left(\bar{\sigma }\right)$ for any $\bar{\delta },\bar{\sigma }\in E_{k}^{W(T)}$ such that $\bar{\delta }\neq \bar{\sigma }$. Denote $T^{*}=J\left(\nu ,T\right)$. Let Γ be a deterministic decision tree for the table $T^{*}$ such that $h\left(\Gamma \right)=h^{d}\left(T^{*}\right)$. Denote by $L_{t}\left(\Gamma \right)$ the number of terminal nodes of Γ. Evidently, $N\left(T\right)\leq L_{t}\left(\Gamma \right)$. One can show that $L_{t}\left(\Gamma \right)\leq k^{h(\Gamma )}$. Therefore, $N\left(T\right)\leq k^{h(\Gamma )}$. Since T≠Λ, N(T)>0. Hence, $h\left(\Gamma \right)\geq \log_{k}N\left(T\right)$. Taking into account that $h\left(\Gamma \right)=h^{d}\left(T^{*}\right)$, we obtain $h^{d}\left(T^{*}\right)\geq \log_{k}N\left(T\right)$.    □

It is not difficult to prove the following upper bound on the minimum cardinality of a separating set for a table by the induction on the number of rows in the table.

**Lemma** **6.** *For any table T from $M_{k}\backslash \left\{\Lambda \right\}$,*

Sep(T)≤N(T)−1.

**Lemma** **7.** *For any complexity measure ψ and any table T from $M_{k}$,*

$$\psi^{a}\left(T\right)\leq \psi^{d}\left(T\right).$$

**Proof.**  Let $T\in M_{k}$. If T=Λ, then $\psi^{a}\left(T\right)=\psi^{d}\left(T\right)=0$. Let $T\in M_{k}\backslash \left\{\Lambda \right\}$. It is clear that each deterministic decision tree for the table *T* is a nondeterministic decision tree for the table *T*. Therefore, $\psi^{a}\left(T\right)\leq \psi^{d}\left(T\right)$.    □

**Lemma** **8.** For any complexity measure ψ and any table T from $M_{k}$, which contains at least two rows, there exists a table $T^{*}\in \left[T\right]$ such that

$$\psi^{a}\left(T^{*}\right)=\psi^{d}\left(T^{*}\right)=W_{\psi }\left(T^{*}\right)=S_{\psi }\left(T^{*}\right)=S_{\psi }\left(T\right).$$

**Proof.**  Let $\bar{\delta }$ be a row of the table *T* such that $S_{\psi }\left(T,\bar{\delta }\right)=S_{\psi }\left(T\right)$. Let *D* be a subset of the set P(T) with the minimum cardinality such that $\psi \left(D\right)=S_{\psi }\left(T,\bar{\delta }\right)$ and in the set of columns labeled with attributes from *D*, the row $\bar{\delta }$ is different from all other rows of the table *T*. Let $\bar{\sigma }$ be the tuple obtained from the row $\bar{\delta }$ by the removal of all numbers that are in the intersection with columns labeled with attributes from the set P(T)\D. Let $\nu :E_{k}^{\left|D\right|}\to E_{2}$ and, for any $\bar{\gamma }\in E_{k}^{\left|D\right|}$, if $\bar{\gamma }=\bar{\sigma }$, then $\nu (\bar{\gamma })=1$ and if $\bar{\gamma }\neq \bar{\sigma }$, then $\nu (\bar{\gamma })=0$. Denote $T^{*}=J\left(\nu ,I\left(P\left(T\right)\backslash D,T\right)\right)$.

From the fact that *D* has the minimum cardinality and from the properties of the function ψ, it follows that for any attribute from the set *D*, there exists a row of *T*, which is different from the row $\bar{\delta }$ only in the column labeled with the considered attribute among attributes from *D*. Therefore, for any attribute of the table $T^{*}$, there exists a row of $T^{*}$, which is different from the row $\bar{\sigma }$ only in the column labeled with this attribute. Thus,

(2) $$S_{\psi }\left(T^{*},\bar{\sigma }\right)=W_{\psi }\left(T^{*}\right).$$

Using properties of the function ψ, we obtain $S_{\psi }\left(T^{*}\right)\leq W_{\psi }\left(T^{*}\right)$. From this inequality and from (2), it follows that

(3) $$S_{\psi }\left(T^{*}\right)=W_{\psi }\left(T^{*}\right).$$

Let Γ be a nondeterministic decision tree for the table $T^{*}$ such that $\psi \left(\Gamma \right)=\psi^{a}\left(T^{*}\right)$, τ be a complete path of Γ such that the row $\bar{\sigma }$ belongs to the table T(τ), and $F\left(\tau \right)=f_{i_{1}}\cdots f_{i_{t}}$. It is clear that $\bar{\sigma }$ is the only row of the table T(τ). Therefore, in columns labeled with attributes $f_{i_{1}},\ldots ,f_{i_{t}}$ the row $\bar{\sigma }$ is different from all other rows of the table $T^{*}$. Thus, $\psi \left(F\left(\tau \right)\right)\geq S_{\psi }\left(T^{*},\bar{\sigma }\right)=W_{\psi }\left(T^{*}\right)$. Therefore, $\psi \left(\Gamma \right)\geq W_{\psi }\left(T^{*}\right)$ and $\psi^{a}\left(T^{*}\right)\geq W_{\psi }\left(T^{*}\right)$. Using Lemmas 1 and 7, we obtain $\psi^{a}\left(T^{*}\right)\leq \psi^{d}\left(T^{*}\right)\leq W_{\psi }\left(T^{*}\right)$. Therefore,

(4) $$\psi^{a}\left(T^{*}\right)=\psi^{d}\left(T^{*}\right)=W_{\psi }\left(T^{*}\right).$$

By the choice of the set *D*, $W_{\psi }\left(T^{*}\right)=S_{\psi }\left(T\right)$. From this equality and from (3) and (4), it follows that $\psi^{a}\left(T^{*}\right)=\psi^{d}\left(T^{*}\right)=W_{\psi }\left(T^{*}\right)=S_{\psi }\left(T^{*}\right)=S_{\psi }\left(T\right)$.    □

It is not difficult to prove the following upper bounds on the minimum complexity of nondeterministic decision trees for a table.

**Lemma** **9.** *For any complexity measure ψ and any table T from $M_{k}$,*

$$\psi^{a}\left(T\right)\leq S_{\psi }\left(T\right)\leq W_{\psi }\left(T\right).$$

## 5. Proofs of Theorems 1, 2 and 3

**Proof** **of** **Theorem** **1.** Since *A* is a closed class, Λ∈A. By definition, $W_{\psi }\left(\Lambda \right)=0$. Using this fact and Lemma 1, we obtain that $F_{\psi ,A}$ is an everywhere defined function and $F_{\psi ,A}\left(n\right)\leq n$ for any n∈ω. Evidently, $F_{\psi ,A}$ is a nondecreasing function. Let T∈A and $W_{\psi }\left(T\right)\leq 0$. Using the positivity property of the function ψ, we obtain T=Λ. Therefore $F_{\psi ,A}\left(0\right)=0$.

(a) Let the functions $S_{\psi }$ and *N* be bounded from above on the class *A*. Then, there are constants a≥1 and b≥2 such that $S_{\psi }\left(T\right)\leq a$ and N(T)≤b for any table T∈A. Let T∈A. Taking into account that *A* is a closed class, we obtain ${\hat{S}}_{\psi }\left(T\right)\leq a$. By Lemma 3, $M_{\psi }\left(T\right)\leq 2a$. From this inequality, inequality N(T)≤b and from Lemma 2, it follows that $\psi^{d}\left(T\right)\leq 2a\log_{2}b$. Denote $c_{0}=2a\log_{2}b$. Taking into account that *T* is an arbitrary table from the class *A*, we obtain that $F_{\psi ,A}\left(n\right)\leq c_{0}$ for any n∈ω.

(b) Let the function $S_{\psi }$ be bounded from above on class *A* and the function *N* be not bounded from above on *A*. Then, there exists a constant a≥2 such that for any table Q∈A,

(5) $$S_{\psi }\left(Q\right)\leq a.$$

Let n∈ω\{0} and *T* be an arbitrary table from *A* such that $W_{\psi }\left(T\right)\leq n$. If $T\in M_{k}C$; then, evidently, $\psi^{d}\left(T\right)=0$. Let $T\notin M_{k}C$. Using the boundedness from below property of the function ψ, we obtain W(T)≤n and S(T)≤a. From these inequalities and Lemma 4, it follows that $N\left(T\right)\leq {\left(kn\right)}^{a}$. From (5) and Lemma 3, it follows that $M_{\psi }\left(T\right)\leq 2a$. Using the last two inequalities and Lemma 2, we obtain

(6) $$\psi^{d}\left(T\right)\leq 2a^{2}\log_{2}n+2a^{2}\log_{2}k.$$

Denote $c_{3}=2a^{2}$ and $c_{4}=2a^{2}\log_{2}k$. Then, $\psi^{d}\left(T\right)\leq c_{3}\log_{2}n+c_{4}$. Taking into account that *n* is an arbitrary number from ω\{0} and *T* is an arbitrary table from *A* such that $W_{\psi }\left(T\right)\leq n$, we obtain that for any n∈ω\{0},

(7) $$F_{\psi ,A}\left(n\right)\leq c_{3}\log_{2}n+c_{4}.$$

Let n∈ω\{0}. Denote $c_{1}=1/\log_{2}k$ and $c_{2}=\log_{k}a$. We now show that

(8) $$F_{\psi ,A}\left(n\right)\geq c_{1}\log_{2}n-c_{2}.$$

Denote $m=\left\lfloor n/a\right\rfloor$. Taking into account that the function *N* is not bounded from above on set *A*, we obtain that there exists a table T∈A such that $N\left(T\right)\geq k^{m}$. Let *C* be a separating set for the table *T* with the minimum cardinality such that $\psi (f_{i})\leq a$ for any $f_{i}\in C$. The existence of such a set follows from the inequality (5) and properties of commutativity and nondecreasing of the function ψ. Evidently, $\left|C\right|\geq m$. Let *D* be a subset of the set *C* such that $\left|D\right|=m$. Denote $T^{0}=I\left(P\left(T\right)\backslash D,T\right)$. One can show that for any attribute of $T^{0}$, there are two rows of the table $T^{0}$ that differ only in the column labeled with this attribute. Therefore, *D* is a separating set for the table $T^{0}$ with the minimum cardinality. By Lemma 6, $N(T^{0})\geq m+1\geq n/a$.

Using Lemma 5, we obtain that there exists a mapping $\nu :E_{k}^{m}\to \omega$ such that

$$h^{d}\left(J\left(\nu ,T^{0}\right)\right)\geq \log_{k}\left(n/a\right).$$

Denote $T^{*}=J\left(\nu ,T^{0}\right)$. Since ψ is a bounded complexity measure, $\psi^{d}\left(T^{*}\right)\geq \log_{k}\left(n/a\right)$. Using the boundedness from above property of the function ψ, we obtain $W_{\psi }\left(T^{*}\right)\leq \left\lfloor n/a\right\rfloor a\leq n$. Hence, the inequality (8) holds. From (7) and (8), it follows that $c_{1}\log_{2}n-c_{2}\leq F_{\psi ,A}\left(n\right)\leq c_{3}\log_{2}n+c_{4}$ for any n∈ω\{0}.

(c) Let the function $S_{\psi }$ be not bounded from above on class *A*. Using Lemma 8, we obtain that the set $D=\{W_{\psi }\left(T\right):T\in A,\psi^{d}\left(T\right)=W_{\psi }\left(T\right)\}$ is infinite. Since the class *A* is closed, Λ∈A, and since $\psi^{d}\left(\Lambda \right)=W_{\psi }\left(\Lambda \right)=0$, 0∈D. Evidently, for any n∈D, $F_{\psi ,A}\left(n\right)\geq n$. Taking into account that $F_{\psi ,A}$ is a nondecreasing function, we obtain that $F_{\psi ,A}\left(n\right)\geq H_{D}\left(n\right)$ for any n∈ω\{0}.    □

**Proof** **of** **Theorem** **2.** Since *A* is a closed class, Λ∈A. By definition, $W_{\psi }\left(\Lambda \right)=0$. Using this fact and Lemma 9, we obtain that $G_{\psi ,A}$ is an everywhere defined function and $G_{\psi ,A}\left(n\right)\leq n$ for any n∈ω. Evidently, $G_{\psi ,A}$ is a nondecreasing function. Let T∈A and $W_{\psi }\left(T\right)\leq 0$. Using the positivity property of the function ψ, we obtain T=Λ. Therefore, $G_{\psi ,A}\left(0\right)=0$.

(a) Let the function $S_{\psi }$ be bounded from above on the class *A*. Then, there is a constant c>0 such that $S_{\psi }\left(T\right)\leq c$ for any T∈A. By Lemma 9, $\psi^{a}\left(T\right)\leq S_{\psi }\left(T\right)\leq c$ for any T∈A. Therefore, for any n∈ω, $G_{\psi ,A}\left(n\right)\leq c$.

(b) Let the function $S_{\psi }$ be not bounded from above on class *A*. Using Lemma 8, we obtain that the set $D=\{W_{\psi }\left(T\right):T\in A,\psi^{a}\left(T\right)=W_{\psi }\left(T\right)\}$ is infinite. Since the class *A* is closed, Λ∈A, and since $\psi^{a}\left(\Lambda \right)=W_{\psi }\left(\Lambda \right)=0$, 0∈D. Evidently, for any n∈D, $G_{\psi ,A}\left(n\right)\geq n$. Taking into account that $G_{\psi ,A}$ is a nondecreasing function, we obtain that $G_{\psi ,A}\left(n\right)\geq H_{D}\left(n\right)$ for any n∈ω\{0}.    □

**Proof** **of** **Theorem** **3.** Let n∈ω and both *n* and n+1 belong to the domain of the function $H_{\psi ,A}$. Immediately from the definition of this function, it follows that $H_{\psi ,A}\left(n\right)\leq H_{\psi ,A}\left(n+1\right)$. Let T∈A and $\psi^{a}\left(T\right)\leq 0$. Using the positivity property of the function ψ, one can show that $T\in M_{k}C$. From Lemma 2, it follows that $\psi^{d}\left(T\right)=0$. Therefore, $H_{\psi ,A}\left(0\right)=0$.

Let $H_{\psi ,A}$ be not an everywhere defined function and *m* be the minimum number from ω such that the value $H_{\psi ,A}\left(m\right)$ is not defined. It is clear that m>0 and the value $H_{\psi ,A}\left(n\right)$ is not defined for each n∈ω such that n≥m. Denote $n_{0}=m-1$. Then, the domain of the function $H_{\psi ,A}$ is equal to $\left\{n:n\in \omega ,n\leq n_{0}\right\}$.

We now consider the case when $H_{\psi ,A}$ is an everywhere defined function.

(a) Let there exist a nonnegative constant c such that $\psi^{d}\left(T\right)\leq c$ for any table T∈A. Then, evidently, $H_{\psi ,A}\left(n\right)\leq c$ for any n∈ω.

(b) Let there be a nonnegative constant *c* such that $\psi^{d}\left(T\right)\leq c$ for any table T∈A. Let us assume that there exists a nonnegative constant d such that $\psi^{a}\left(T\right)\leq d$ for any table T∈A. Then, the value $H_{\psi ,A}\left(d\right)$ is not defined, but this is impossible. Therefore, the set $D=\{\psi^{a}\left(T\right):T\in A\}$ is infinite. Since Λ∈A, 0∈D. By Lemma 7, $\psi^{a}\left(T\right)\leq \psi^{d}\left(T\right)$ for any table T∈A. Hence, $H_{\psi ,A}\left(n\right)\geq n$ for any n∈D. Taking into account that $H_{\psi ,A}$ is a nondecreasing function, we obtain that $H_{\psi ,A}\left(n\right)\geq H_{D}\left(n\right)$ for any n∈ω.    □

## 6. Conclusions

In this paper, we studied the complexity of deterministic and nondeterministic decision trees for tables from closed classes of conventional decision tables. The obtained results allow us to point out the cases when the complexity of deterministic and nondeterministic decision trees is essentially less than the complexity of the set of attributes attached to columns of the table. This may be useful in applications. Future research will be devoted to a more in-depth study of relationships between the complexity of deterministic and nondeterministic decision trees for conventional decision tables from closed classes.

## Data Availability

Not applicable.

## References

[1] Breiman L., Friedman J.H., Olshen R.A., Stone C.J. (1984). Classification and Regression Trees.

[2] Chikalov I., Lozin V.V., Lozina I., Moshkov M., Nguyen H.S., Skowron A., Zielosko B. (2013). Three Approaches to Data Analysis—Test Theory, Rough Sets and Logical Analysis of Data.

[3] Fürnkranz J., Gamberger D., Lavrac N. (2012). Foundations of Rule Learning.

[4] Pawlak Z. (1991). Rough Sets—Theoretical Aspects of Reasoning about Data.

[5] Quinlan J.R. (1993). C4.5: Programs for Machine Learning.

[6] Rokach L., Maimon O. (2007). Data Mining with Decision Trees—Theory and Applications.

[7] Moshkov M. (2005). Time Complexity of Decision Trees. Trans. Rough Sets.

[8] Moshkov M., Zielosko B. (2011). Combinatorial Machine Learning—A Rough Set Approach.

[9] Moshkov M. (2020). Comparative Analysis of Deterministic and Nondeterministic Decision Trees.

[10] Boros E., Hammer P.L., Ibaraki T., Kogan A. (1997). Logical analysis of numerical data. Math. Program..

[11] Boros E., Hammer P.L., Ibaraki T., Kogan A., Mayoraz E., Muchnik I.B. (2000). An Implementation of Logical Analysis of Data. IEEE Trans. Knowl. Data Eng..

[12] Pawlak Z., Skowron A. (2007). Rudiments of rough sets. Inf. Sci..

[13] Molnar C. (2022). Interpretable Machine Learning. A Guide for Making Black Box Models Explainable.

[14] Blum M., Impagliazzo R. (1987). Generic Oracles and Oracle Classes (Extended Abstract). Proceedings of the 28th Annual Symposium on Foundations of Computer Science.

[15] Buhrman H., de Wolf R. (2002). Complexity measures and decision tree complexity: A survey. Theor. Comput. Sci..

[16] Hartmanis J., Hemachandra L.A. (1987). One-way functions, robustness, and the non-isomorphism of NP-complete sets. Proceedings of the Second Annual Conference on Structure in Complexity Theory, Cornell University.

[17] Tardos G. (1989). Query complexity, or why is it difficult to separate *NP^A^*∩*coNP^A^* from *P^A^* by random oracles *A*?. Combinatorica.

[18] Moshkov M., Markov A.A. (1989). On depth of conditional tests for tables from closed classes. Combinatorial-Algebraic and Probabilistic Methods of Discrete Analysis.

[19] Ostonov A., Moshkov M. (2023). Comparative analysis of deterministic and nondeterministic decision trees for decision tables from closed classes. arXiv.

[20] Ostonov A., Moshkov M. (2023). Deterministic and strongly nondeterministic decision trees for decision tables from closed classes. arXiv.

[21] Post E. (1941). Two-Valued Iterative Systems of Mathematical Logic.

[22] Robertson N., Seymour P.D. (2004). Graph Minors. XX. Wagner’s conjecture. J. Comb. Theory, Ser. B.

[23] Moshkov M., Yablonskii S.V. (1983). Conditional tests. Problemy Kibernetiki.

